# Three-Dimensional Gastrointestinal Organoid Culture in Combination with Nerves or Fibroblasts: A Method to Characterize the Gastrointestinal Stem Cell Niche

**DOI:** 10.1155/2016/3710836

**Published:** 2015-11-30

**Authors:** Agnieszka Pastuła, Moritz Middelhoff, Anna Brandtner, Moritz Tobiasch, Bettina Höhl, Andreas H. Nuber, Ihsan Ekin Demir, Steffi Neupert, Patrick Kollmann, Gemma Mazzuoli-Weber, Michael Quante

**Affiliations:** ^1^II. Medizinische Klinik, Klinikum rechts der Isar, Technische Universität München, 181675 München, Germany; ^2^Chirurgische Klinik und Poliklinik, Klinikum rechts der Isar, Technische Universität München, 281675 München, Germany; ^3^Lehrstuhl für Humanbiologie, Technische Universität München, 385350 Freising, Germany

## Abstract

The gastrointestinal epithelium is characterized by a high turnover of cells and intestinal stem cells predominantly reside at the bottom of crypts and their progeny serve to maintain normal intestinal homeostasis. Accumulating evidence demonstrates the pivotal role of a niche surrounding intestinal stem cells in crypts, which consists of cellular and soluble components and creates an environment constantly influencing the fate of stem cells. Here we describe different 3D culture systems to culture gastrointestinal epithelium that should enable us to study the stem cell niche *in vitro* in the future: organoid culture and multilayered systems such as organotypic cell culture and culture of intestinal tissue fragments *ex vivo*. These methods mimic the *in vivo* situation *in vitro* by creating 3D culture conditions that reflect the physiological situation of intestinal crypts. Modifications of the composition of the culture media as well as coculturing epithelial organoids with previously described cellular components such as myofibroblasts, collagen, and neurons show the impact of the methods applied to investigate niche interactions *in vitro*. We further present a novel method to isolate labeled nerves from the enteric nervous system using Dclk1-CreGFP mice.

## 1. Introduction

Adult tissue stem cells are necessary for tissue homeostasis and regeneration after injury, but under certain conditions they may undergo malignant transformation and give rise to cancer. This hypothesis has been widely studied* in vivo* but* in vitro* methods to analyze the underlying factors and the interaction of the epithelium and stroma are missing. A common and distinct feature of most adult stem cells is their localization [1], as they reside within a specific and protected anatomical location called stem cell niche. Such a niche is composed of cellular components surrounding stem cells, extracellular matrix (ECM), and soluble factors. It is thought that the primary function of this stem cell niche is stem cell retention with controlled symmetric and asymmetric divisions. Anchorage of stem cells is mediated by their contact with the ECM and relies on adherens junctions [2, 3]. The cellular part of the niche contains stromal cells such as the osteoblastic cells in bone marrow, Sertoli cells in germ line stem cell niche, or pericryptal fibroblasts in the intestine [1, 4]. As the gastrointestinal epithelium is prone to inflammation and carcinogenesis, it is important to decipher regulatory mechanisms of the intestinal stem cell niche not only in physiology, but also during inflammation and carcinogenesis.

The intestine is an organ with a high epithelial cell turnover comprising a self-renewal every two to seven days in the context of normal tissue homeostasis [5], making it a perfect model to study the impact of the niche on proliferation and differentiation of stem cells. This plasticity originates from the presence of multipotent intestinal stem cells (ISC), which reside in crypts or gastrointestinal glands [5].* Lgr5*-positive (and* Prom1-*/*Sox9*-positive) rapid cycling stem cells reside at the crypt base, hence also called crypt base columnar cells (CBC), and the four cell lineages of the intestinal crypt were shown to be derived from these stem cells [6]. Surrounded by endothelial cells (vascular and lymphatic endothelium), pericytes, cells of the immune system, fibroblasts, and neurons, ISCs are constantly modulated by the release of cytokines, chemokines, growth factors, and neurotransmitters and may thereby be modified towards a carcinogenic fate [7, 8]. The maintenance and proliferation of stem cells is highly dependent on modulations of signaling pathways such as Wnt, Notch, BMP, or Hedgehog [8]. Recently, Paneth cells were shown to be an important component of the intestinal stem cell niche [9]; however depletion of this cell type in mouse did not alter significantly the crypt phenotype [10]. That would suggest that niche factors are provided by stromal cells.

A great majority of* in vitro* studies in biomedical research are dominated by the application of two dimensional (2D) cell culture models. However, the latter poorly reflect cellular heterogeneity and cellular behavior of tissues* in vivo*. Moreover, 2D cell culture does not allow studying the communication between different cell types or ECM-cell interaction. In the biomedical research there is growing interest for the application of three-dimensional (3D) cell culture systems. Recently, the 3D culture of mouse intestinal crypts has been developed, which is known as minigut culture or organoid culture [11]. The crypt culture recapitulates the cellular diversity of the intestinal epithelium. Furthermore, in the intestinal organoid culture crypt and villus domains can be identified, thus resembling proliferating and differentiated compartments in the intestine* in vivo*. As it is possible to add stromal cells to the organoid culture, it is a promising physiologically relevant model to study the niche factors* ex vivo*. Here we propose to study the niche factors utilizing nerves and fibroblasts as niche cells for intestinal and cardia crypt organoids* in vitro*.

## 2. Methods

### 2.1. Animals

Animal experiments were approved by the local committee of animal welfare. For SI crypt isolation tissue from C57BL/6 mice (Jackson laboratories) was used. For the isolation of intestinal myofibroblasts wild type C57BL/6J mice at the age of 4 weeks–2 years were used. Dclk1-CreGFP mice were crossed to Rosa26-mTomato/mGFP (R26-TGFP) reporter strains as previously described [12]. Mice employed for neuronal coculture were aged between 7 and 10 days and transgenic ubiquitously expressing the protein* Dclk1Cre* in* Dclk1*-positive cells and their lineages, combined with the reporter protein-complex Tom^fl/fl^GFP. To obtain Barrett organoid cardia cells, tissues from pL2-IL-1b mice at the age of one year were isolated. The pL2-IL-1beta mice carry the EBV-L2-IL-1*β* transgene, overexpressing the human IL-1beta in the esophageal and squamous forestomach mucosa. The mice show esophagitis which progresses to metaplasia and dysplasia at an older age (~12 months) [13]. Additionally, for cardia and intestinal crypt culture Lgr5-CreTMG mice with an inducible Cre and stable GFP expression in Lgr5-positive cells at the age of 60 and 32 weeks respectively, were employed [6].

### 2.2. Intestinal Organoid Culture (Intestinal Crypts)

The small intestine (SI) of mice was taken out and washed in PBS (Life Technologies), removing all fat and adjacent tissue. It was opened longitudinally and washed in PBS to remove all remaining food residues. The SI tissue was cut into ca. 3–5 cm long parts, which were stored in a petri dish containing cold PBS + 10% fetal bovine serum (FBS) (Life Technologies), referred to later as PBS/FBS. The intestine parts were flattened on a petri dish and the villi were scraped off using a cover glass. This step was repeated on the inside and outside of the SI parts. These SI parts were cut with a scalpel into 2–4 mm pieces and transferred into 20 mL PBS/FBS in a 50 mL falcon tube. The tissue samples were allowed to sediment and the supernatant was removed. Additional 10 mL of PBS/FBS was added to wash the tissue and the supernatant was removed. These steps were repeated until the supernatant was clear (approx. 5 times). The remaining tissue was digested in PBS + EDTA (2 mM) for 15 min at 4°C on a shaker. After digestion the supernatant was removed. The tissue was resuspended in 10 mL PBS/FBS and filtered through a 70 *μ*m cell strainer (BD Biosciences) into a new 50 mL falcon tube. This step was repeated several times (~4 times). To pellet the crypts they were centrifuged for 8 min, 800 rpm at 4°C. The supernatant was removed as completely as possible, 150–200 *μ*L Matrigel was added to the pellet avoiding air bubbles. 50 *μ*L Matrigel was plated in every well of a prewarmed 24-well plate. In every well 0.5 mL of crypt culture medium (CM) was added, which is composed of advanced DMEM/F12 (Life Technologies), supplemented with serum-free B27 (1 : 50, Life Technologies), N2 (1 : 100, Life Technologies), N-acetylcysteine (50 mM, Sigma), recombinant murine epithelial growth factor (EGF 50 ng/mL, Peprotech), Noggin (100 ng/mL, Peprotech), R-Spondin (1 *μ*g/mL, Peprotech), Glutamax-I Supplement (1 : 100, Life Technologies), Penicillin/Streptomycin (500 *μ*g/mL, Life Technologies), and HEPES (10 *μ*M, Life Technologies) (see Table 1). The cultures were incubated in a humidified incubator at 37°C and 5% CO_2_.

After 1 day, growth of the isolated crypts can be monitored. After 2-3 days SI crypts start budding, resembling the crypt-villus structure in the intestine. The medium was changed every second day. Approximately every week the crypts can be split 1 : 2 to 1 : 4, depending on the number and size of the crypts. To passage the organoids, the medium was replaced by 0.5 mL ice-cold medium (CM without growth factors, also described as complete medium), pipetted up and down a few times to disrupt the Matrigel, and put in a new 15 mL falcon. Disrupted crypts should no longer be visible by eye. They were pelleted at 800 rpm for 5 min at 4°C and the appropriate amount of Matrigel was added to the pellet. The Matrigel drops are plated again into a 24-well plate and the crypt culture medium was added.

### 2.3. Isolation of Intestinal Myofibroblasts

Intestinal myofibroblasts were isolated using the explant technique. The murine small intestine was harvested and cut into 2-3 mm fragments. The tissue fragments were washed in a cold washing solution that was composed of Hanks' Balanced Salt Solution (HBSS) (Life Technologies), 1% Penicillin/Streptomycin (Life Technologies), and 50 *μ*g/mL Gentamicin (Life Technologies) and incubated in 1 mM Dithiothreitol (DTT) for 15 min at room temperature. Afterwards, the tissue fragments were incubated in 1 mM EDTA for 30 min at 37°C with occasional stirring. After washing with HBSS, incubation with 1 mM EDTA was repeated. Then the tissue fragments were washed and incubated with 1 mg/mL collagenase type I (Sigma) for 30 min at 37°C. After washing with a solution containing HBSS, 10% FBS (Life Technologies), and 1% Penicillin/Streptomycin, the tissue fragments were plated and cultured in a medium containing RPMI1640 (Life Technologies), 10% FBS, 1% Penicillin/Streptomycin, and 100 *μ*g/mL Normocin (Invivogen). The myofibroblasts migrate out of the tissue fragments and adhere to the culture plate.

### 2.4. Crypt-Fibroblast Coculture in Organotypic Cell Culture System (OTC)

Myofibroblasts and crypts were isolated as stated above from 6-week–8-month-old mice (*n* = 3 for the 2-day cultures and *n* = 4 for the 7-day cultures), expanded, and then used for the experiment. Acellular and cellular matrix were prepared as previously published [14], with minor modifications. 900 *μ*L of acellular matrix was pipetted on a transwell (24 mm transwell with 3.0 *μ*m pore polycarbonate membrane insert, Corning), which was placed into a 6-well transwell carrier (Organogenesis). After the solidification, 450 *μ*L of cellular matrix containing intestinal myofibroblasts was poured. After the stabilization of the matrix, 450 *μ*L of cellular matrix containing intestinal crypts was poured on the top. Per well 7.5 mL medium containing RPMI1640 (Life Technologies), 10% FBS (Life Technologies), 1% Penicillin/Streptomycin (Life Technologies), and 330 ng/mL R-Spondin (Peprotech) were added. At day 0 cells were seeded. R-Spondin was added to the medium only at day 0. At days 2 and 7 the culture was fixed in formalin, dehydrated, and embedded in paraffin. On the cut paraffin slides H&E, Ki-67 (Abcam), and *α*-SMA (Abcam) staining were performed. Total number of crypts was quantified on slides that were stained for H&E. Disintegrated crypts were counted as nonviable. Survival was defined as percentage of viable crypts.

### 2.5. Tissue Culture* Ex Vivo*

Small intestine was harvested from 5-week-old mice, cut into small pieces, embedded in a matrix, and cultured. The method was performed as previously published [15], with exception of the composition of the acellular bottom layer and cell-containing layer, which were prepared according to the protocol for organotypic cell culture [14]. The culture was performed in a transwell (24 mm transwell with 3.0 *μ*m pore polycarbonate membrane insert, Corning) that was placed into a 6-well transwell carrier (Organogenesis). The culture medium was composed of Ham's F12 (Life Technologies), 20% FBS (Life Technologies), and 50 *μ*g/mL Gentamicin (Life Technologies) and it was exchanged after one week. 1 mL of medium was added per well. After two weeks, the culture was fixed in formalin and embedded in paraffin and PAS staining was performed.

### 2.6. Influence of the Extracellular Matrix on Phenotype of Intestinal Organoids

Small intestinal crypts from three wild type 7-week–17-month-old mice were isolated and cultured in Matrigel as described above. For the experiment, crypts were seeded either in Matrigel or in a cellular matrix, which was prepared as previously published [14] with the exception of FBS, which was not added. The crypts were mixed with the cellular matrix and 50 *μ*L of matrix was seeded per well in a 24-well plate. Crypts were seeded at day 0 and the culture medium was the same as previously published [16]. Analysis and fixation in formalin were performed at day 2. Sections were stained for Ki-67 (Abcam).

### 2.7. Cardia Organoid Culture

The harvested stomach was opened along the large curvature and flushed with cold PBS + 10% FBS to remove food residues. To isolate the tissue of the squamocolumnar junction (SCJ), the opened stomach was put flat on a hard surface inside out; the tissue of interest was dissected, washed in PBS, chopped into small pieces, and transferred into 20 mL PBS buffer substituted with EDTA and ethylene glycol tetraacetic acid (EGTA, final concentration 2 mM). To detach the individual tissue layers, the harvested tissue was incubated for 45 min on a shaker at 4°C. Following incubation the supernatant was removed and 10 mL PBS with 10% FBS was added. Analogous to the isolation of intestinal crypts, further disruption of the tissue was accomplished using the shearing forces of pipetting. The fragments were passed through a 70 *μ*m cell strainer (BD Biosciences). The cell suspension was spun down at 600 rpm for 5 min. The pellet was resuspended in Matrigel and seeded onto a preheated 24-well plate with drops of 50 *μ*L Matrigel in each well. For long-term cultures, the organoids were cultured in Wnt-conditioned intestinal crypt culture medium (0.5 mL per well), which was added freshly every second day. Wnt-CM (later referred to as cardia medium, CaM) was derived from L-Wnt3a cell line (ATCC) that was cultured in the medium composed of advanced DMEM/F12 (Life Technologies), HEPES (10 *μ*M, Life Technologies), Penicillin/Streptomycin (500 *μ*g/mL, Life Technologies), and Glutamax-I Supplement (1 : 100, Life Technologies). Cardia organoids typically need Wnt3a for growth and long-term survival as an additional growth factor [17]. For efficient expansion of the organoids, they have to be passaged after 7 and 10 days in culture with the same procedure as intestinal organoids. The cultures were fixed in formalin at day 10, dehydrated, and embedded in paraffin. After cutting paraffin slides, hematoxylin and eosin (H&E), periodic acid–Schiff (PAS), Ki-67 (Abcam), and *α*-SMA (Abcam) staining were performed.

### 2.8. Isolation of Neuronal Tissue and Coculture

Neuronal tissue is derived from the myenteric plexus by removing the seromuscular layer of the prepared small intestine (adapted from [18, 19]). During the isolation process, isolated tissue was kept on ice in minimum essential medium (MEM, Life Technologies). The obtained tissue was digested for ca. 45–80 min at 37°C in HBSS containing collagenase type II (1 *μ*g/*μ*L, Worthington). Using a microscope, the digestion process was carefully monitored for its progress. After the first incubation period, insufficiently digested plexi were separated and digested for another 15 min. The obtained small fragments were then carefully disrupted using injection syringes (Sterican 23 G × 1  1/4′′ or 20 G × 1  1/2′′). To stop the digestion process HBSS supplemented with 10% FBS was added. The cell suspension was centrifuged (600 rpm, 5 min) and, after another washing step with HBSS/FBS, resuspended in advanced DMEM/F12 (Life Technologies). For growth and differentiation of neurons, cells were plated in Matrigel together with cardia organoids and cultured in neurobasal complete medium (NBM), which is composed of neurobasal medium (Life Technologies), nerve growth factor (NGF, 1 : 1000, Life Technologies), serum-free B27 (1 : 50, Life Technologies), N2 (1 : 100, Life Technologies), N-acetylcysteine (50 mM, Sigma), recombinant murine epithelial growth factor (EGF 50 ng/mL, Peprotech), Noggin (100 ng/mL, Peprotech), R-Spondin (1 *μ*g/mL, Peprotech), Glutamax-I Supplement (1 : 100, Life Technologies), Penicillin/Streptomycin (500 *μ*g/mL, Life Technologies), and HEPES (10 *μ*M, Life Technologies). Medium was changed every other day. Cocultures were incubated in a humidified incubator at 37°C and 5% CO_2_.

### 2.9. Calcium Imaging

Ca^2+^-imaging allows visualizing alterations of intracellular [Ca^2+^]_i_ visible by using fluorescent dyes that change the intensity of their fluorescence depending on the [Ca^2+^]_i_. Although the direct contribution of Ca^2+^ to the neuronal membrane potential is limited, it has been shown that membrane potential events are closely linked to changes in [Ca^2+^]_i_ [20, 21]. Therefore the Ca^2+^ indicator Fluo-4 AM (Invitrogen) was used to detect activation of the neurons in the cocultures. The cells were incubated in 10 *μ*M Fluo-4 AM for 20 minutes, followed by a 20 min wash out. During the Ca^2+^-imaging experiments cells were continuously perfused with Krebs solution [22] at 37°C, which was constantly fumigated with carbogen (95% oxygen and 5% CO_2_). To ensure a neuronal staining that was stable for several hours we added 1.25 mM probenecid (Sigma) to the Krebs solution used for the experiments. Probenecid is an inhibitor of organic anion transporters eliminating dyes and indicators from the cytoplasm [23]. The changes in fluorescence were determined using a digital camera (AxioCam Hsm, Zeiss) and the Zeiss Axio Vision software in combination with an inverted microscope (Axio Observer A1, Zeiss). The frame rate of the recording was 2 Hz, which is sufficient to reveal neuronal activation. For viability testing we applied 100 *μ*M nicotine (Sigma) via pressure application (PDES-2L, npi electronic GmbH, Tamm, Germany) through a pulled glass pipette (tip diameter ca. 5 *μ*m). Nicotine acts as an agonist at the nicotinergic acetylcholine receptor and therefore is suitable as a viability test for cultured enteric neurons [24]. To quantify the changes in fluorescence upon nicotinic activation of acetylcholine receptors, we assigned the resting light intensity (RLI) in the regions of interest.

### 2.10. Reverse Transcription Polymerase Chain Reaction (RT-PCR)

RNA was isolated using the RNeasy Mini Kit (Qiagen). Residual genomic DNA was eliminated by DNase digestion on the column (Qiagen). cDNA was generated by the Reverse Transcription Kit (Promega). The primers applied were *α*-SMA (fw 5′ CGCTGTCAGGAACCCTGAGA 3′, rv 5′ ATGAGGTAGTCGGTGAGATC 3′), *β*-actin (fw 5′ CCCTGAACCCTAAGGCCAACC 3′, rv 5′ ACCCCGTCTCCGGAGTCCATC 3′), E-cadherin (fw 5′ ACCACTGCCCTCGTAATCGAA 3′, rv 5′ CGTCCTGCCAATCCTGATGAA 3′), Lgr5 (fw 5′ GACGCTGGGTTATTTCAAGTTCAA 3′, rv 5′ CAGCCAGCTACCAAATAGGTGCTC 3′), villin 1 (fw 5′ GACGTTTTCACTGCCAATACCA 3′, rv 5′ CCCAAGGCCCTAGTGAAGTCTT 3′), and vimentin (fw 5′ AACACCCGCACCAAC 3′, rv 5′ TCCGGTACTCGTTTGACT 3′).

### 2.11. Organoid/Enteroid Evaluation and Statistical Analysis

Proliferation and growth of organoids can be evaluated* in vitro* in several ways; we here applied the determination of the diameter and the circumference of the organoids per passage and compared the means on a timeline. The cardia organoids were measured at day 7 and day 10 using Axio Vision software (Zeiss) and ImageJ. Evaluation of the growth of intestinal crypts in organotypic cell culture system was based on the analysis of H&E staining: diameter of crypts after 2 and 7 days of culture was measured. The statistical analysis was performed using GraphPad Prism. Comparing of two groups was performed using an unpaired two-tailed *t*-test. A standard deviation of *P* < 0.05 was statistical significant.

## 3. Results

### 3.1. 3D Cell Culture Systems to Study the Stem Cell Niche in the Gastrointestinal Tract

In order to analyze the stem cell niche* in vitro* we analyzed three different* in vitro* culture systems to ideally mimic the* in vivo* situation and still be able to understand the impact of the different cellular and acellular factors.

**Figure 1:**
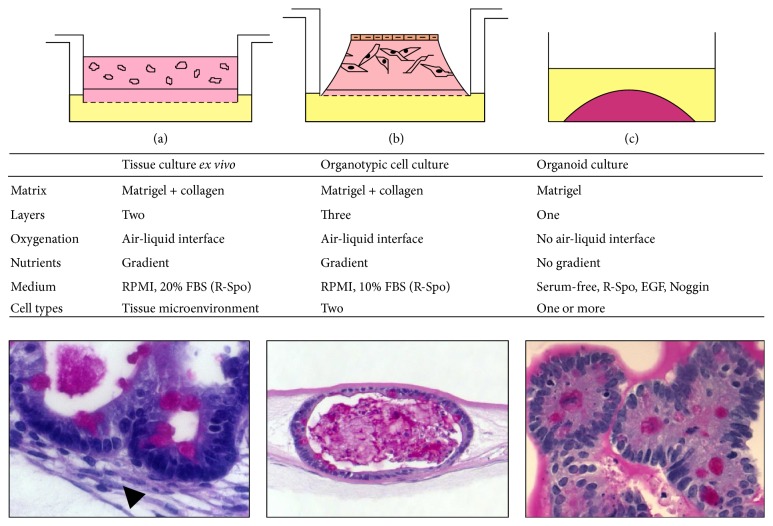
Summary of three-dimensional methods to culture intestinal epithelium. (a) Tissue culture* ex vivo*. Arrowhead indicates stromal cells. (b) Organotypic cell culture. (c) Organoid culture. PAS staining. R-Spo, R-Spondin.

The tissue culture* ex vivo *system is based on embedding whole tissue fragments in a matrix. Here we modulated a previously published tissue culture* ex vivo *system [15], utilizing an additional acellular layer and a cell-containing layer that were prepared according to the protocol for the originally published OTC system [14] (Figure 1(a)). The culture was performed in transwells as described above. Firstly an acellular layer was poured, which mimics the basement membrane. Then a cellular layer containing tissue fragments was poured. The medium was added only to the outer dish, which created an air-liquid interface and leads to better oxygenation of the culture. Of note, addition of R-Spondin (which is crucial for organoid culture) was not necessary; however we observed increased efficiency of growing epithelium after the addition of R-Spondin (not shown), similarly as it was previously reported [15]. Although this type of culture preserves a native stromal niche (indicated by arrowhead, Figure 1(a)) and can be analyzed after formalin fixation and paraffin embedding by immunohistochemistry, it does not represent a direct tool to analyze epithelial-stromal interaction* in vitro *without the use of genetically modified mouse models and certainly not for human material.

In contrast to the tissue culture* ex vivo* both OTC and organoid culture enable combining and therefore analyzing distinct cell types. In the OTC system epithelial and stromal cells are embedded in separate matrix layers. Since multilayer and multicellular OTC systems [14] seem to provide gradients of nutrients (feeding from the bottom) and they seem to mimic the structure of an organ much better than organoid cultures, we tested the suitability of OTC as a novel model to study the intestinal stem cell niche with murine intestinal crypts isolated from B6 WT mice. We cultured intestinal crypts in a multilayer OTC system (Figure 1(b)) similarly to the tissue culture* ex vivo*; the culture was performed in a transwell. Firstly, the acellular layer was poured; then a cellular layer with intestinal myofibroblasts was added. On the top, another cellular layer containing crypts was placed. Medium was added only to the outer dish (feeding from the bottom) thus enabling an air-liquid interface with crypts occasionally being directed to the open surface.

In contrast, in the enteroid/organoid culture cells are embedded in Matrigel and cultured in a “Matrigel drop” (Figure 1(c)). Culture medium contains growth factors such as R-Spondin, EGF, and Noggin, which are necessary to maintain growth and structure of the crypts. It has been defined that crypts from the intestine should be named enteroids as soon they form a budding “minigut” and only the combination of these cells with other cell types allows the term organoids. The combination of other cell types such as fibroblasts and nerves with the enteroid system mimics a specific* in vivo* situation and can be called organoid [25]. Although the organoid culture represents a physiologically relevant cell culture system and it has been shown that small intestinal crypts in an organoid culture system contain a crypt and villus domain [11], it has limitations. The organoid culture system does, for example, not recapitulate the gradient of nutrients, which is present along crypt-villus axis* in vivo*.

### 3.2. Characterization of Small Intestinal Organoids and Small Intestinal Myofibroblasts

**Figure 2:**
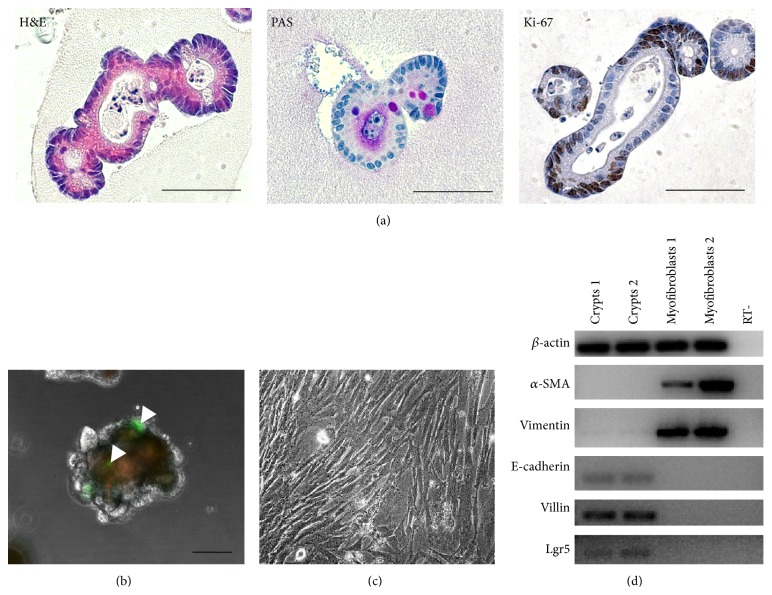
Characterization of small intestinal organoids and small intestinal myofibroblasts used to reconstruct intestinal stem cell niche* ex vivo*. (a) H&E staining, PAS staining, and Ki-67 staining of small intestine organoids. Scale bar, 50 *μ*m. (b) Lgr5-GFP-positive epithelial cells in cultured conditions (marked with arrowheads). Scale bar, 100 *μ*m. (c) Morphology of small intestine myofibroblasts, phase contrast microscopy. (d) Confirmation of the purity of small intestine organoids and small intestine myofibroblasts by Reverse-Transcription PCR. RT- (reaction without the addition of reverse transcriptase) served as the negative control.

In order to characterize small intestinal organoids, the culture was fixed in formalin, embedded in paraffin, and stained. H&E staining showed that an intestinal organoid is composed of a monolayer of polarized columnar epithelial cells (Figure 2(a), left). Moreover small intestinal organoids contain lumen and are characterized by the presence of buds. PAS staining revealed presence of mucus producing cells and the secretion of mucus into the lumen (Figure 2(a), middle). Besides differentiated zones characterized, for example, by the presence of mucus producing cells, in small intestinal organoids proliferative zones can be distinguished as can be seen from Ki-67 staining (Figure 2(a), right). Cells with proliferative activity seemed not to be randomly distributed, but rather accumulated in areas where buds grew (Figure 2(a), right). Moreover, small intestinal organoids expressed Lgr5, a marker of intestinal stem cells (Figure 2(b)), and we demonstrate Lgr5-positive cells isolated from an Lgr5 CreTM mouse in which all LgR5 expressing cells also have GFP expression in the intestinal organoids (Figure 2(b)). Myofibroblasts isolated from small intestine and cultured* in vitro* were elongated and spindle-like (Figure 2(c)). To confirm the lineage identity of small intestinal organoids and small intestinal myofibroblasts, RT-PCR for the epithelial cell markers E-cadherin and villin as well as mesenchymal cell markers such as *α*-SMA and vimentin was performed. The data revealed that small intestinal organoids were positive for E-cadherin and villin and negative for *α*-SMA and vimentin, thus confirming their epithelial lineage identity (Figure 2(d)). Small intestinal myofibroblasts expressed *α*-SMA and vimentin and were negative for epithelial cell markers, thus confirming their stromal cell identity (Figure 2(d)).

### 3.3. Fibroblasts Improve Organoid Culture Survival

**Figure 3:**
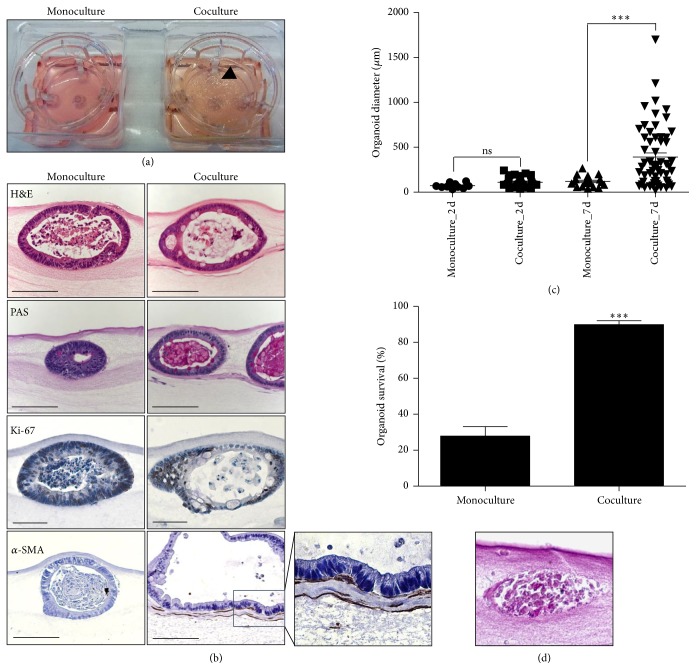
Fibroblasts improve organoid growth and organoid culture survival. (a) Macroscopic image of the crypt monoculture and coculture in an organotypic cell culture system at day 7. (b) Characterization of the crypt monoculture (left panel) and coculture (right panel) in an organotypic cell culture system at day 7 by immunohistochemistry staining: H&E (scale bar 100 *μ*m), PAS (scale bar 100 *μ*m), Ki-67 (scale bar 50 *μ*m), and *α*-SMA staining (scale bar 100 *μ*m), with adjacent magnification. (c) Organoid diameter measured on H&E slides from crypt monoculture and coculture in organotypic cell culture system after 2 and 7 days of culture. Each dot represents a single organoid. *P* < 0.0001 (one-way ANOVA with Bonferroni comparison). (d) Lower, example of a nonviable crypt in organotypic cell culture system, H&E staining. Scale bar, 25 *μ*m. Upper, survival of crypts in an organotypic cell culture system, for the monoculture 169 crypts were quantified; for the coculture 213 crypts were quantified. *P* < 0.0001, two-tailed *t*-test.

To study the role of fibroblasts as a niche factor for ISCs, small intestinal crypts were cultured together with intestinal myofibroblasts in an OTC system (Figure 1(b)) and compared to the crypt monoculture. For the monoculture, the same matrix was used but with the only change that the myofibroblasts were not added to the cellular layer. After 7 days of the cultivation we observed increased macroscopic visibility of the coculture when compared to the monoculture (Figure 3(a)). Both crypt OTC monoculture and crypt OTC coculture contained mucus producing cells and proliferating cells as demonstrated by PAS and Ki-67 staining (Figure 3(b)). Proliferating cells were not homogenously distributed, but proliferating compartments could be distinguished especially in the coculture. *α*-SMA staining confirmed the presence of underlying spindle-shape myofibroblasts in the crypt OTC coculture (Figure 3(b), with adjacent magnification). To check whether the myofibroblasts influence crypt growth over the time, the diameter of crypts at day 2 and day 7 was measured from cultures that were fixed in formalin, embedded in paraffin, and stained for H&E. Although we did not detect significant changes in crypt size between monoculture and coculture at day 2, we observed that at day 7 crypt diameter in the monoculture was ~100 *μ*m, whereas in the coculture it was ~400 *μ*m (Figure 3(c)), suggesting that myofibroblasts improve growth of the crypts. To further investigate the influence of myofibroblasts on the crypts, analysis of the organoid survival was performed, which relied on the quantification of viable crypts based on the H&E staining. A viable crypt was defined as a crypt with intact epithelial cell monolayer, while a nonviable crypt was defined as an accumulation of cellular detritus (Figure 3(d), lower). In the crypt OTC monoculture 30% of crypts were viable, whereas in the crypt OTC coculture 90% of crypts were viable (Figure 3(d), upper), indicating that myofibroblasts improve organoid culture survival and thus supporting the hypothesis that myofibroblasts provide some niche factors for ISCs.

### 3.4. Collagen Modulates the Phenotype of Intestinal Crypts

**Figure 4:**
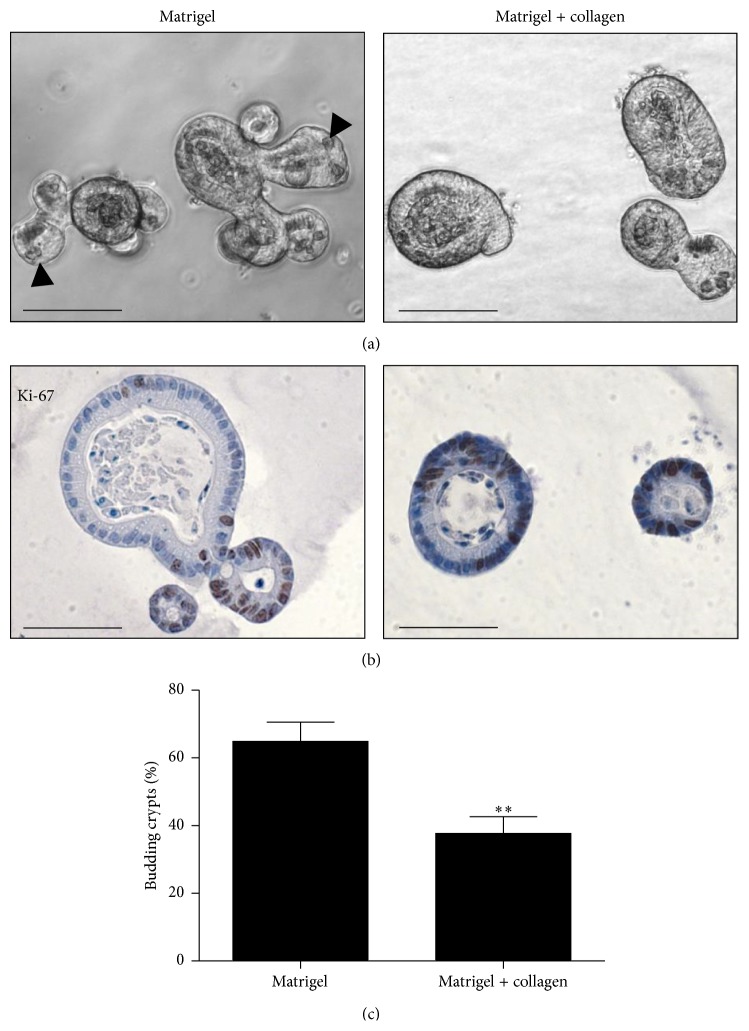
Collagen modulates the phenotype of small intestine organoids. (a) Morphology of small intestine organoids cultured in the presence of EGF, Noggin, and R-Spondin. Arrowheads indicate buds. Scale bar, 100 *μ*m. (b) Ki-67 staining of small intestine organoids, scale bar, 50 *μ*m. (c) Collagen reduces budding of small intestine organoids. In total 1700–1800 crypts were quantified per condition. *P* = 0.0045, two-tailed *t*-test.

Interestingly, crypts cultured in the OTC system almost did not have buds, which are a characteristic feature of 3D intestinal tissue cultures, as shown by crypts that were cultured in the organoid system (Figure 4(a)). Since the extracellular matrix was shown to alter the phenotype of normal mammary epithelium [26] and given that matrix composition of OTC differs from that one used in organoid culture of intestinal crypts, we hypothesized that the extracellular matrix might contribute to shaping the phenotype of the normal intestinal epithelium. To test this hypothesis, we utilized the organoid culture system and embedded crypts in the OTC cellular matrix. To exclude the potential effect of an air-liquid interface and the effect of serum, the culture was performed in a 24-well plate (identically as the organoid culture) [11, 27] and without addition of serum into the cellular matrix. In control conditions (crypts seeded in Matrigel) approximately 70% crypts contained buds, while in the OTC cellular matrix only 40% crypts were budding (Figure 4(c)), suggesting that collagen reduces the formation of buds. Although crypts in OTC cellular matrix showed a decreased number of buds, they contained proliferating cells, as revealed by Ki-67 staining (Figure 4(b)).

### 3.5. Isolating Distinct Neurons from the Enteric Nervous System for* In Vitro* Culture

As described above, the nervous system is thought to have an important function in intestinal epithelial stem cell regulation and in the development of neoplasia [12, 28, 29]. Upon surgical or pharmacological denervation of the stomach, our collaborators and we recently demonstrated that carcinogenesis in the denervated part of the stomach was depending on innervation [17]. At the same time, this was correlated with a downregulation of Wnt3a in the stem cell niche of the denervated part of the stomach.

**Figure 5:**
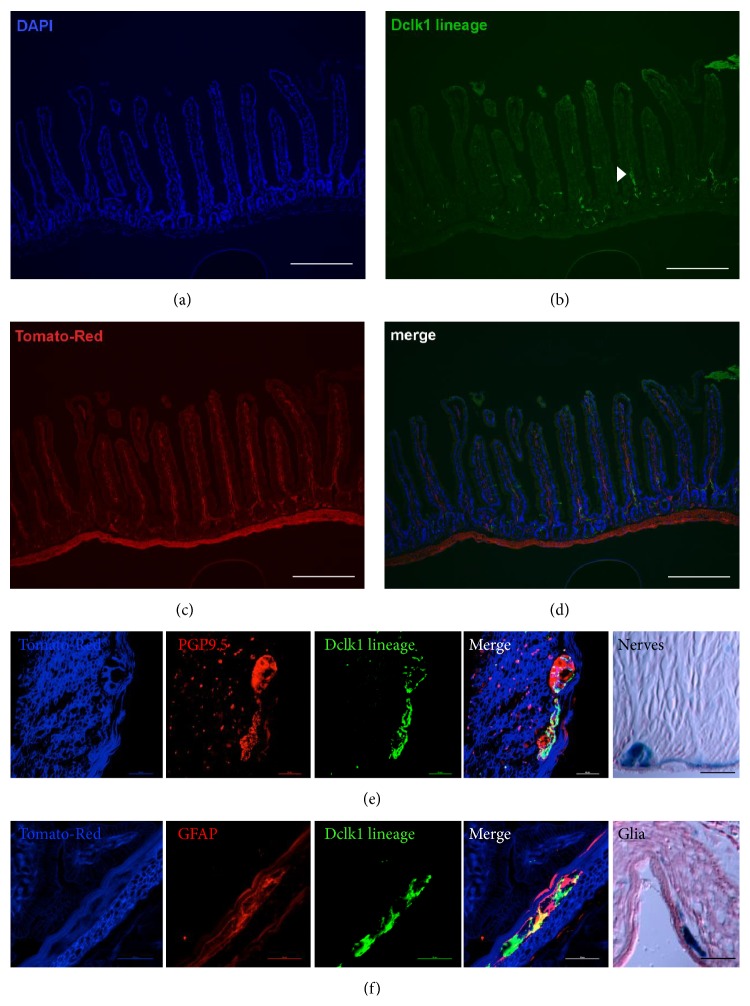
Positive Dclk1 lineage shown in the enteric nervous system with GFP labeled neurons. Immunofluorescence of small intestine from* Dclk1.Cre.GFP-Tom* ^*fl/fl*^ *GFP* mice. (a) DAPI for cell nuclei. (b) Positive GFP-fluorescence in neurons (*Dclk1.Cre.GFP-Tom* ^*fl/fl*^ *GFP* label recombined cells of the Dclk1 lineage from birth on) invading organoids and single Dclk1-positive cells in epithelium, arrowhead indicating lineage of Dclk1-positive neurons, (c) Tom-Red ubiquitously expressed as marker protein complex. (d) Merge of (a)–(c), scale bars 100 *μ*m. (e) Cultured neurons identified by positive GFP-fluorescence and overlapping with PGP9.5 staining as proof of lineage, last panel demonstrating lacZ stained neuronal structures next to smooth musculature within the GI tract, scale bar, 50 *μ*m. (f) Overlapping fluorescence with GFAP as proof of lineage of glia within myenteric plexus, last panel showing lacZ stained glia within myenteric plexus, scale bar, 50 *μ*m.

We recently generated a BAC transgenic mouse line that expresses constitutive Cre-recombinase under the control of the Dclk1 gene locus [12]. We subsequently crossed Dclk1-Cre mice to a Tomato/GFP reporter mouse line (B6.129S4-Gt(ROSA)26Sortm4(ACTB-tdTomato-EGFP)Luo/J), in which membrane targeted Tomato (RFP) is replaced by membrane targeted Green fluorescent protein (GFP) upon Cre-mediated recombination. Interestingly, in this mouse line with constitutive active Cre expression from embryogenesis on, we observed lineage tracing in nerve-like structures throughout the entire gut (Figures 5(a)–5(d)). Within the Dclk1 lineage, we frequently observed enteric GFAP+ glial cells and PGP9.5+ nerves and ganglia (Figures 5(e)–5(f)), suggesting that the Dclk1 lineage labels parts of the enteric nervous system (ENS), which has been shown to modulate responses to injury [30].

Subsequently, we established a neuron-organoid coculture model as a novel method to examine the role of nerves on the intestinal stem cell niche [12] in health and disease such as Barrett esophagus, for which we also recently developed a new mouse model [13], which allows us to isolate mouse Barrett epithelial organoids. Using our robust organoid culture model, we cultured and cocultured organoids from Barrett epithelium of the mouse in different media as described above.

**Figure 6:**
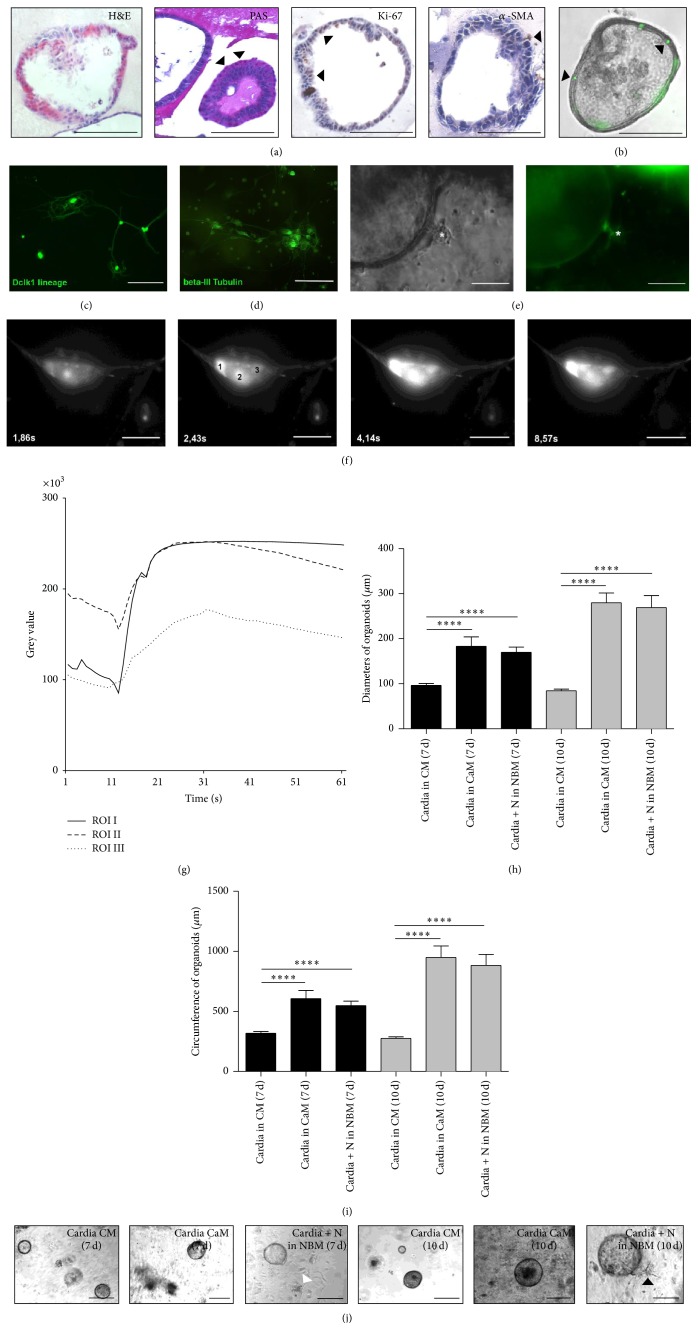
Cardia organoid cultures and cocultures with neurons as a model to investigate the gastrointestinal stem cell niche. (a) IHC for H&E, PAS, Ki-67, and *α*-SMA for further characterization of the method, arrowheads indicating positive stained cells, scale bars, 100 *μ*m. (b) Light microscopy of Lgr5-GFP-positive organoid, scale bar, 50 *μ*m. (c) Immunofluorescence of Dclk1-labeled neurons in cultured conditions (NBM). (d) Cultured neurons (NBM) stained with beta-III Tubulin, scale bar, 100 *μ*m. (e) Cocultured neuron with cardia organoid, light microscopy and immunofluorescence,  ^*∗*^Dclk1-labeled neuron (green fluorescence) neighboring crypt wall, scale bar, 100 *μ*m. (f) Ca-imaging of neuron in cocultured conditions (NBM), different points in time beginning from application of nicotinic acid to Fluo-4-labeled neurons, brightening of neuron indicating Ca-influx. Scale bar, 50 *μ*m. (g) Analysis of the RLI of stimulated neuron, ROI I, ROI II, and ROI III measuring different regions of the neuron as indicated in (f), picture 2 (regions labeled 1, 2, and 3). ((h), (i)) Analysis of organoids and cocultured neurons in different conditions, distribution of mean diameters and circumferences per group and point in time and standard error of the mean (SEM) (CM = crypt medium, CaM = Wnt-conditioned cardia medium, NBM = cocultured neurons in neurobasal complete medium, groups compared at 7 d and 10 d), measured in *μ*m; comparison of average organoid growth between groups applying two-tailed *t*-test ( ^*∗*^level of statistical significance, ^*∗∗∗∗*^ *P* < 0.0001), *n* = 3 experiments per group and point in time. (j) Representative light microscopic image of each group and point in time (arrowheads indicating neurons), scale bar, 100 *μ*m.

Similar to the characterization of the cultured small intestinal crypts, H&E staining showed that cardia organoids are composed of a monolayer of polarized columnar epithelial cells (Figure 6(a)). Moreover the organoids contain lumen and are characterized by the presence of mucus producing cells and the secretion of mucus into the lumen (Figure 6(a), PAS staining). Besides differentiated zones characterized, for example, by the presence of mucus producing cells, in cardia organoids proliferative zones also can be distinguished as can be seen from Ki-67 staining (Figure 6(a)). Cells with proliferative activity seemed not to be randomly distributed, but rather accumulated in expanding areas of the organoids (Figure 6(a)). To verify the purity of the isolated cardia organoids, *α*-SMA showed to be predominantly negative except only few positive fibroblasts adjacent to proliferative epithelium (Figure 6(a)). To prove the presence of stem cells in our cardia organoids, we demonstrate the presence of Lgr5-CreTM-GFP labeled epithelial cells in cultured conditions (see Figure 6(b)).

For isolation of neurons from the enteric nervous system, 7–10-day-old pups were used from* Dclk1.Cre.GFP-Tom* ^*fl/fl*^ *GFP* mice, as described above (Figure 6(c)). This figure demonstrates the pure isolation of neuronal structures from the gut, which then could be cultured with cardia organoids from the Barrett model. As additional proof of nerves showing the positive lineage, a beta-III Tubulin staining was performed of neurons cultured* in vitro*, which almost exclusively labeled neurons (Figure 6(d)). Using the Dclk1 lineage reporter we were therefore able to specifically isolate ENS neurites and grow them in 3D coculture conditions (Figure 6(e)). Neurons showed prominent neurite outgrowth and were physiologically active till day 10 of culture as demonstrated by utilizing Ca-imaging (Figures 6(f) and 6(g)). The analysis of the RLI showed an abrupt increase upon stimulation of neurons with nicotine, subsequently showing a decrease of RLI over time in the regions of interest (ROI) in the absence of nicotine (Figure 6(g)).

### 3.6. Neurons Enhance Growth of Murine Cardia Organoids

Comparing different conditions of organoid cultures with and without neurons and variations in the culture media, we observed significant differences between the cultures with and without neurons as described previously [12]. We compared organoid cultures of Barrett epithelium isolated from Barrett esophagus from pL2-IL-1b mice [13] cultivated with CM, Wnt-conditioned CaM, and the coculture with neurons in NBM, the normal organoid cultures in CM being the control groups at seven days and ten days (for composition of media, see Table 1). The comparison of cardia organoids in Wnt-conditioned medium and cocultured with neurons remained of special interest, as the role of nerves and the Wnt signaling was recently investigated intensively in gastrointestinal tumorigenesis [17].

Interestingly, the means ± SEM (standard error of the mean) of the groups showed highly significant differences already after seven days of culture (see Table 2), in total three experiments per group and point in time were performed. The size of the organoids cultivated in CaM (Wnt-conditioned medium) compared to CM showed significantly larger organoids after seven and ten days applying both methods of measuring organoid size (*P* < 0.0001). The same result is shown for the comparison of organoids in CM compared to organoids in NBM with neurons in coculture (NBM + N) (*P* < 0.0001). Organoids in NBM cocultured with neurons compared with organoids cultured in CaM did not show any significant differences in size at both day 7 and day 10 (Figures 6(h) and 6(i), examples of cultured and cocultured organoids shown in Figure 6(j)). Interestingly, the organoid count over time reached a 1.5–2-fold increase in number of organoids per well already at day 4 of culture in both CaM conditions and cocultures with nerves compared to the control group cultured in CM (data not shown), although organoid count balanced at day 10 of culture for all conditions compared. Additionally, judging from the analysis of the diameters and circumferences, the latter are feasible to calculate from the diameters (Table 2).

## 4. Conclusions

Here we describe the utilization and modification of distinct 3D culture methods that can be used to analyze the role and function of different stem cell niche components* in vitro*. The basic element of each 3D cell culture is a matrix, which plays a scaffolding role for the cells and tissues, similarly to ECM that is present in organs* in vivo*. Examples of the matrices used in 3D cell biology are the following: Matrigel, collagen, and Hydrogel [31–35]. 3D cell cultures have shown altered gene expression profiling when compared to 2D [36]. Here we describe the modification and establishment of three distinct 3D cell culture methods to study the adult gastrointestinal epithelium in order to evaluate their potential to study niche factors: (1) tissue culture* ex vivo*, (2) organotypic cell culture (OTC), and (3) organoid culture (see Figure 1). We further performed cocultures with adult mouse fibroblasts, collagen, or adult murine neurons, and our data show that cellular components of an* in vitro* niche, represented here by myofibroblasts and neurons, support growth of epithelial organoids that contain stem cells; however the precise mechanisms of interaction may be subject to further investigation.

The tissue culture* ex vivo *is characterized by the highest biological complexity from all of these methods. It preserves stem cells and their native niche and any manipulations in tissue culture* ex vivo* will affect the whole stem cell niche unit. Nevertheless, this culture method does not allow a specific analysis of distinct components of the stem cell niche and therefore we provide here a novel aspect to split those different components such as adult pericryptal fibroblasts, collagen, and adult nerves from the ENS into distinct separate coculture assays. Both OTC and organoid culture fulfill these criteria. The advantage of OTC compared to organoid culture, however, is the feature of an air-liquid interface as recently described as a cellular differentiation factor [37], and handling for the immunohistochemistry purposes, even given the fact that OTC requires a lot of cells. In the adult mouse intestine, the epithelium is surrounded by a layer of subepithelial myofibroblasts [38] and likely the heterogeneity of myofibroblasts correlates with the gradient of crucial niche factors (e.g., Wnt ligands, BMPs) that determine the proliferation and differentiation zones in the crypt-villus unit [39]. Based on their anatomical location, subepithelial myofibroblasts were hypothesized to be a cellular component of the intestinal epithelial niche and to regulate self-renewal and differentiation of ISCs [40]. Cocultures in which cells of human origin were utilized have shown that a certain type of infant fibroblasts can increase the longevity of human organoid/enteroid cultures [33, 34]. Here we demonstrate that adult murine pericryptal fibroblasts do also affect crypt size and survival in an organotypic culture setting with a direct epithelial mesenchymal cell contact, suggesting again that fibroblasts represent niche cells for intestinal stem cell and their differentiation capacities.

As another example, we demonstrate that normal intestinal crypts exhibit reduced budding when cultured in matrix composed of collagen and Matrigel (cellular matrix used for OTC). Since Matrigel already contains collagen, the cellular matrix for OTC is characterized by an increased concentration of collagen that is likely leading to an increased stiffness of the matrix. Our results confirm previous findings that collagen can modify the matrix of the organoid culture [41] and suggest that increased collagen concentration has a major impact on the growth conditions of intestinal organoids as it restricts formation of buds and should therefore be considered as a caveat in future analysis of organoid culture systems.

Accumulating studies have pointed to the importance of nerves in the regulation of the intestinal stem cell niche [12, 17, 42, 43]. A role for nerves in the development of neoplasia has also been postulated [17, 28, 29]. Nerve fibers were shown to support carcinogenesis by either mechanical aspects, being an anatomical structure used as guidance for tumor growth by perineural tumor invasion, or functional aspects, comprising neurotransmitters influencing local vascularization, migratory activity of neighboring cells, or the neoneurogenesis itself [29, 44, 45]. Interestingly, upon surgical or pharmacological denervation of the stomach, it was shown that the denervated part of the stomach had a markedly reduced tumor incidence and progression in different mouse models of gastric cancer [17]. At the same time, this was correlated with a downregulation of Wnt3a in the stem cell niche of the denervated part of the stomach. Here we present a novel method to isolate distinct enteric nerves from the mouse ENS identified by the Dclk1-Cre-Tom/GFP lineage and perform cocultures of nerves with cardia organoids* in vitro* (Figures 5 and 6).

We previously demonstrated that intestinal [12] and gastric [17] organoids can be cocultured with neurons. Here we demonstrate that when culturing cardia organoids, that specifically depend on Wnt signaling in the 3D monoculture system, upon coculture with nerves these are apparently able to replace Wnt signaling. The Wnt pathway represents a fundamental signaling pathway for ISCs and dysregulations of this pathway lead to intestinal cancer [46]. Our analysis of cultures and cocultures of predominantly circular cardia organoids from our Barrett esophagus mouse model demonstrates some interesting details about the interaction of neurons within the intestinal niche. Organoids cultured in Wnt-conditioned medium compared to standard crypt medium already showed a significant difference of organoid growth after seven and ten days (see Table 2, Figures 6(h) and 6(i)), applying both the measurement of diameters and circumferences for growth determination. The coculture with neurons also showed a highly significant difference of organoid growth compared to standard crypt medium after seven and ten days without any supplement of Wnt. The same effect is reflected in the 1.5–2-fold increase in organoid count per well in both Wnt-conditioned monoculture and neuronal coculture compared to standard monoculture. Interestingly, there was no significant difference in growth and organoid count between organoids cultured in Wnt-conditioned medium or cocultured with neurons, neither at day 7 nor at day 10. Using calcium imaging we showed that the cocultured neurons were alive and physiologically active during the observation period, which is underlined by these significant differences of organoid growth between control and cocultured group.

Judging from these results, in the absence of Wnt it is most likely the neuronal influence on organoids that promotes organoid growth, which may implicate that neurons and their transmitters replace the effect of Wnt signaling. This argues for a neuronal signaling, which creates a proliferative environment and supports organoid growth stronger than the usually applied growth factors used in standard crypt medium, which was already postulated and shown by Zhao and colleges to possibly exist [17]. In their study, gastric organoid growth was markedly promoted if cocultured with neurons and without neurons but upon stimulation with pilocarpine (an unspecific muscarinic receptor agonist), and Wnt target genes such as* Lgr5*,* Cd44*, and* Sox9* were shown to be elevated [17]. In future studies it remains to be elucidated which factors lead to an increase of organoid growth in the neuronal coculture compared to Wnt-conditioned medium. Neurons are rich sources of neurotrophic factors including nerve growth factor (NGF), GDNF family of ligands (such as GDNF, artemin, and neurturin), or transforming growth factor beta 1 [47]. It is imaginable that these factors may substantially promote the maintenance and growth of organoids or of the stem cell niche in the stomach. In our experiments we applied NGF within the neurobasal medium, so that it remains to be clarified if the effect we describe here is due to this single neurotrophic factor or others. Our observations also imply that enteric neurons may have similar trophic functions in the maintenance of epithelial integrity in the gut, as it is known for enteric glia cells [48]. Thus we strongly support future emphasis on investigating pathways of the interaction between neurons and intestinal stem cells to characterize the composition of the cancer stem cell niche, fostering the development of therapeutics directing the origin of malignancy as putative therapeutic targets.

**Figure 7:**
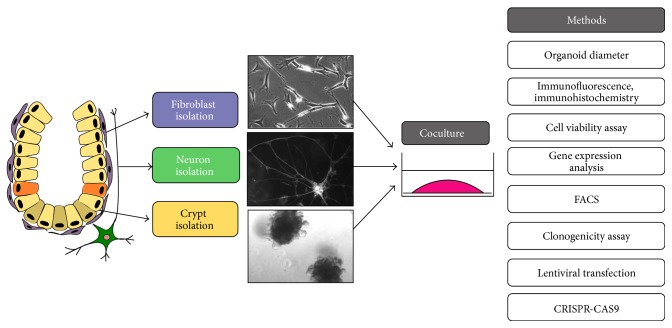
Reconstruction of stem cell niche in the intestine* in vitro* and proposed workflow. Intestinal crypts and stromal cells (neurons and/or myofibroblasts) are isolated and combined together in three-dimensional system such as organoid culture. Role of stromal cells as stem cell niche can be analyzed, for example, by the measurement of organoid diameter, cell viability assay, clonogenicity assay, gene expression analysis, and different types of staining. Identification of stem cell markers and individual cell types can be analyzed by FACS. In addition, genetic manipulations in organoids can be performed by lentiviral transfection and CRISPR-CASP9 system.

Currently, a broad spectrum of downstream methods is available in order to analyze the organoids, for example, immunohistochemistry and immunofluorescence [11, 49], the CRISP-Cas9 system [50, 51], organoid transduction with retroviral construct [52], or a clonogenicity assay [53] (see Figure 7). In combination with the culture of intestinal fragments* ex vivo *[15], in which the specific local niche is preserved, we suggest these types of cultures as reliable physiologically relevant 3D models to analyze a mesenchymal-epithelial cross talk in the intestinal stem cell niche and identify underlying mechanism of early tumorigenesis. Although our results so far do not allow any basic conclusions on the specific role of these components in a stem cell niche, the presented coculture methods point to an important function of stromal cell types in the described 3D culture conditions.

## Figures and Tables

**Table 1:** Composition of standard media applied in the described methods.

Culture conditions	Composition
Crypt medium (CM)	Advanced DMEM/F12 + HEPES + Pen/Strep + Glutamax + growth supplements (B27, N2) + ENR (EGF, R-Spondin, and Noggin) + N-acetylcysteine

Cardia medium (CaM)	Wnt-conditioned advanced DMEM/F12 + HEPES + Pen/Strep + Glutamax + growth supplements (B27, N2) + ENR (EGF, R-Spondin, and Noggin) + N-acetylcysteine

**Table 2:** Measurements of organoid size and circumference at 7 d and 10 d in *µ*m, *n* = 3 experiments.

	Mean ± SEM	Mean ± SEM	*n*
	(Diameter)	(Circumference)	(Measurements)
Cardia in CM (7 d)	96.54 ± 4.316	318.5 ± 14.67	93
Cardia in CaM (7 d)	183.3 ± 20.74	606.8 ± 66.98	79
Cardia + N in NBM (7 d)	169.7 ± 11.76	548.3 ± 37.37	96
Cardia in CM (10 d)	84.40 ± 3.850	275.8 ± 12.44	77
Cardia in CaM (10 d)	279.9 ± 21.67	949.4 ± 94.87	60
Cardia + N in NBM (10 d)	269.1 ± 26.72	883.2 ± 90.80	79

SEM = standard error of the mean.
